# Author Correction: Guangxi cobra venom-derived NGF promotes the osteogenic and therapeutic effects of porous BCP ceramic

**DOI:** 10.1038/s12276-019-0270-y

**Published:** 2019-07-05

**Authors:** Pan Jin, Fuqiang Yin, Li Huang, Li Zheng, Jinmin Zhao, Xingdong Zhang

**Affiliations:** 1 Guangxi Engineering Center in Biomedical Materials for Tissue and Organ Regeneration, The First Affiliated Hospital of Guangxi Medical University, Nanning, People’s Republic of China; 2 Guangxi Collaborative Innovation Center for Biomedicine, The First Affiliated Hospital of Guangxi Medical University, Nanning, People’s Republic of China; 3 Guangxi Key Laboratory of Regenerative Medicine, The First Affiliated Hospital of Guangxi Medical University, Nanning, People’s Republic of China; 4 The Medical and Scientific Research Center, Guangxi Medical University, Nanning, People’s Republic of China; 5 National Engineering Research Center for Biomaterials, Sichuan University, Chengdu, People’s Republic of China

**Correction to: Experimental & Molecular Medicine**

10.1038/emm.2016.173 published online 7 April 2017

After online publication of this article, the authors noticed an error in the Figure section. The correct statement of this article should have read as below.

In the article cited above, incorrect figure was placed in Fig. 2c, d. The corrected image of Fig. 2 is printed below. Other parts of this article remain unchanged.

**Fig. 2:**
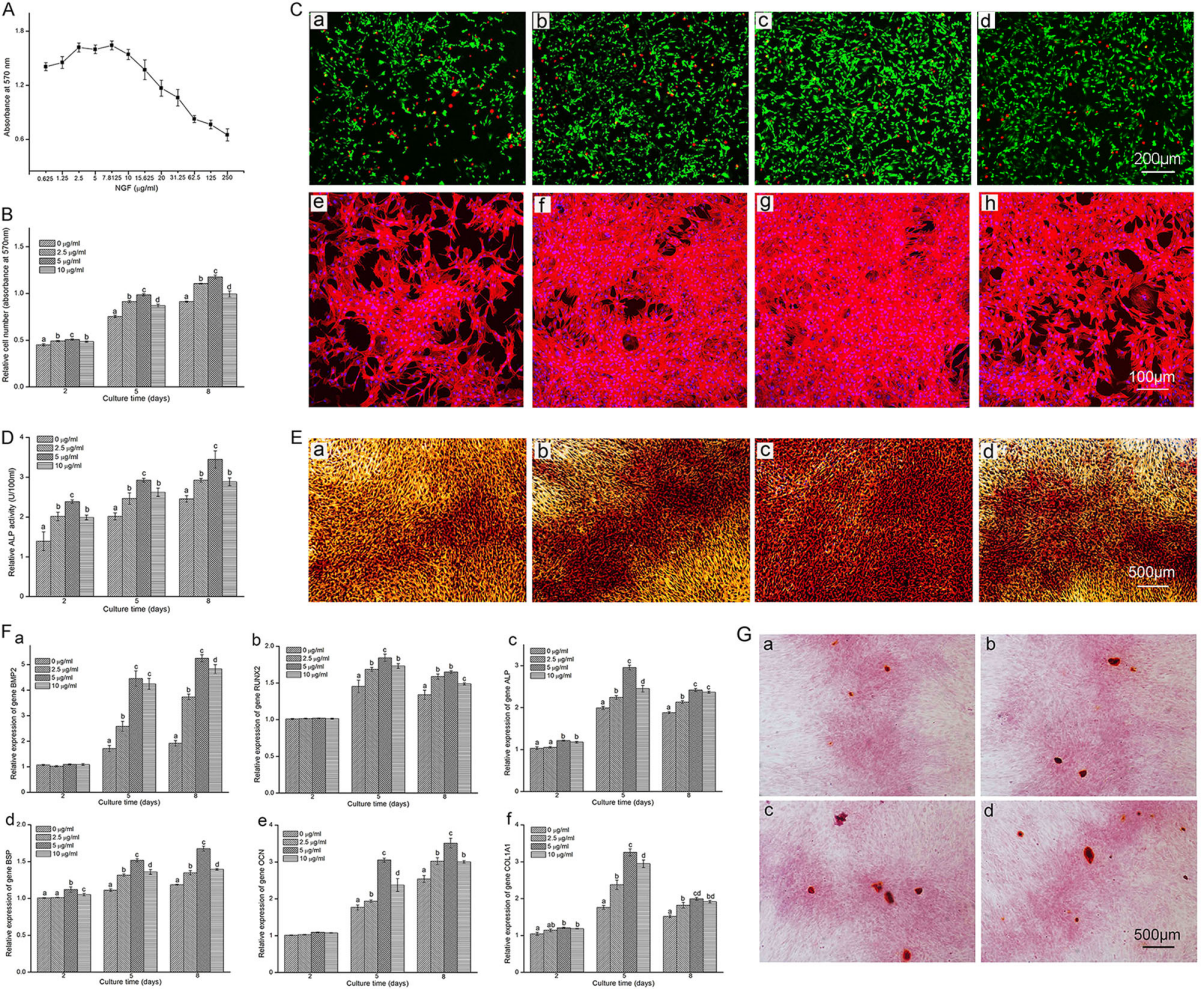
Concentration screening, cytotoxicity assay, FDA-PI staining, rhodamine phalloidin-Hoechst 33258 staining, alkaline phosphatase (ALP) activity assay, ALP staining, osteogenic-specific gene expression and Alizarin red staining of monolayer-cultured osteoblasts. (**A**) Screening of nerve growth factor (NGF) using various concentrations (0.625, 1.25, 2.5, 5, 7.8125, 10, 15.625, 20, 31.25, 62.5, 125 and 250 μg ml^−1^) on 2D cultured osteoblasts using the MTT method after 3 days of treatment (*n* = 3). (**B**) Cytotoxicity assay with NGF at 0, 2.5, 5 and 10 μg ml^−1^ on days 2, 5 and 8 (*n* = 9). (**C** (**a**–**d**)) FDA-PI staining of osteoblasts treated with NGF at 0, 2.5, 5 and 10 μg ml^−1^ after 8 days of treatment (Scale bar = 200 μm). (**C** (**e**–**h**)) Rhodamine phalloidin- Hoechst 33258 staining of a monolayer culture treated with 0, 2.5, 5 and 10 μg ml^−1^ NGF after 8 days (Scale bar = 100 μm). (**D**) ALP activity assay of osteoblasts treated with 0, 2.5, 5 and 10 μg ml^−1^ NGF on days 2, 5 and 8 (*n* = 9). (**E** (**a**–**d**)) ALP staining of osteoblasts treated with 0, 2.5, 5 and 10 μg ml^−1^ NGF after 8 days (Scale bar = 500 μm). (**F** (**a**–**f**)) Relative expression of bone morphogenetic protein-2 (*BMP2*, **F**(**a**)), runt-related transcription factor 2 (*RUNX2*, **F**(**b**)), alkaline phosphatase (*ALP*, **F**(**c**)), bone sialoprotein (*BSP*, **F**(**d**)), osteocalcin (*OCN*, **F**(**e**)) and alpha-1 type I collagen (*COL1A1*, **F**(**f**)) in osteoblasts treated with 0, 2.5, 5 and 10 μg ml^−1^ NGF on days 2, 5 and 8 (*n* = 3). (**G** (**a**–**d**)) Alizarin red staining of osteoblasts treated with 0, 2.5, 5 and 10 μg ml^−1^ NGF after 8 days (Scale bar = 500 μm). The values are presented as the means ± standard deviation. Bars with different letters at the same time point are significantly different from each other at *P* < 0.05, and bars with the same letter exhibit no significant difference

The authors apologize for any inconvenience caused.

